# How Materials and Device Factors Determine the Performance: A Unified Solution for Transistors with Nontrivial Gates and Transistor–Diode Hybrid Integration

**DOI:** 10.1002/advs.202104896

**Published:** 2021-12-16

**Authors:** Chuan Liu, Xiaojie Li, Yiyang Luo, Ya Wang, Sujuan Hu, Chenning Liu, Xiaoci Liang, Hang Zhou, Jun Chen, Juncong She, Shaozhi Deng

**Affiliations:** ^1^ State Key Laboratory of Optoelectronic Materials and Technologies and the Guangdong Province Key Laboratory of Display Material and Technology, School of Electronics and Information Technology Sun Yat‐Sen University Guangzhou 510006 China; ^2^ School of Electronic and Computer Engineering Peking University Shenzhen Graduate School Shenzhen 518055 China

**Keywords:** charge transport, field‐effect transistors, hybrid integration

## Abstract

Advanced field‐effect transistors (FETs) with nontrivial gates (e.g., offset‐gates, mid‐gates, split‐gates, or multi‐gates) or hybrid integrations (e.g., with diodes, photodetectors, or field‐emitters) have been extensively developed in pursuit for the “More‐than‐Moore” demand. But understanding their conduction mechanisms and predicting current–voltage relations is rather difficult due to countless combinations of materials and device factors. Here, it is shown that they could be understood within the same physical picture, i.e., charge transport from gated to nongated semiconductors. One proposes an indicator based on material and device factors for characterizing the transport and derives a unified and simplified solution for describing the current–voltage relations, current saturation, channel potentials, and drift field. It is verified by simulations and experiments of different types of devices with varied materials and device factors, employing organic, oxide, nanomaterial semiconductors in transistors or hybrid integrations. The concise and unified solution provides general rules for quick understanding and designing of these complex, innovative devices.

## Graphical abstract

Advanced field‐effect transistors with nontrivial gates (e.g., offset‐gates, mid‐gates, split‐gates, or multi‐gates) or hybrid integrations (e.g., with diodes, photodetectors, or field‐emitters) have been difficult to understand due to countless combinations of materials. A concise and unified solution is given and validated for understanding how various materials and device factors determine the device performance.

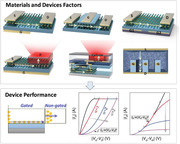

## 1 Introduction

**Figure 1:**
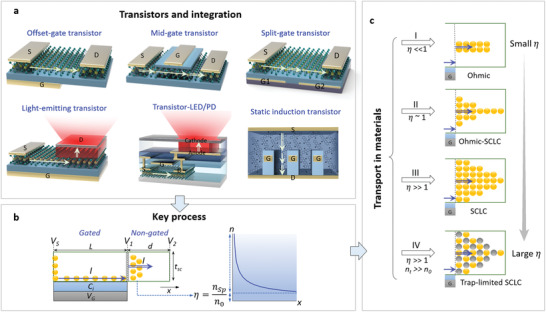
From devices to transport in materials. a) Transistors and integration containing coupled channels. The lateral structures are on the first row and the vertical structures are in the lower row. b) The key physical process, i.e., charges from gated to non‐gated semiconductors. Potentials, current, length, and thickness parameters are marked. c) Transport mechanisms in materials. The single‐line arrows and double‐line arrows indicate the transport in the gated and nongated semiconductors, respectively. In (c), the yellow and grey dots represent the movable and trapped carriers, respectively, and the number of them illustrates the density in the *x*‐direction.

Field‐effect transistors (FETs) or thin‐film transistors (TFTs) with non‐trivial gate structures have been explored for superior functions in cutting‐edge research, including the offset‐gate structure, mid‐gate structure, split‐gate structure, light‐emitting transistors, or other multigate or partial‐gate structures.^[^ ^1^, ^2^, ^3^ ^]^ A significant feature is that gated and nongated semiconductor channels are coupled together. Such coupled channels are also critical to integrate sensing, memory, computing, or illumination by integrating transistors with photodiodes (PDs),^[^ ^4^ ^]^ light‐emitting diodes (LEDs),^[^ ^5^ ^]^ field emitters,^[^ ^6^ ^]^ sensors,^[^ ^7^ ^]^ and etc. Some of them are illustrated in **Figure** **1**a, which are made with a broad range of new semiconductors including organic small molecules or polymers, metal oxide, low dimensional semiconductors, perovskites, and etc.^[^ ^8^, ^9^, ^10^, ^11^ ^]^ However, the countless possibilities of material combination and interface make it difficult to understand the relationship between material and device parameters and device characteristics. So far, these devices have been comprehended or explained case by case in experiments only through complex and special models and nonuniversal numerical simulation.^[^ ^8^, ^11^, ^12^ ^]^ Therefore, a concise physical picture and a general framework for understanding such new devices or predicting their characteristics are still blank.

A general physical framework would answer both fundamental and practical questions: Why and what material or device factors will affect the charge transport and current conduction mechanisms? How do these factors determine the current–voltage relations and the current saturation? How is the electric potential and field distributed in the channels? How to design the device structures? The framework should also be as concise and universal as possible, so that it can be applied to devices and semiconductors with various particularities. The target of this study is to understand the mechanisms and predict the performance of those new devices made of various materials.

Here, we focus on the key physical process, i.e., charge carriers drift from the gated‐channel into a nongated channel (Figure 1b). First, we derive a basic theory framework with simple and universal equations for describing how charge transport occurs, how current–voltage relations behave, and how the electric potential and field evolve. Then, we verify the theory framework by simulating an offset‐gate transistor as an example. Finally, we apply it to examine different experimental devices and briefly discuss how to quickly understand, calculate, analyze, and design these devices.

## 2 The Physical Picture

From Gauss law and continuity principle, the current on the boundary between the gated and nongated semiconductor is continuous (Figure 1b, the gray boundary). In gated semiconductors, the electric resistance (*R*) is related to the drain current (*I*_D_) by using the gradual channel approximation:^[^ ^13^ ^]^ d*V*  = *I*_D_ d*R*  = *I*_D_d*x*/*WμC*_i_[*V*_G_ − *V*_th_ − *V*(*x*)] . Here, *W* is the channel width, *C*_i_ is the capacitance per unit area for the insulating dielectric layer, *V*_G_ is the gate voltage, *V*_th_ is the threshold voltage, and *V*(*x*) is the local potential. Denote *V*_S_ as the source potential as transistors with organic or low‐dimensional semiconductors usually have injection barriers, unlike silicon MOSFETs. Denote *V*_1_ as the potential at the end (|*V*_S_|< |*V*_1_| ≤ |*V*_G_ ‐ *V*_th_|) and *L* as the gated channel length. Integrate d*V* from *V*_S_ to *V*_1_ and d*x* from 0 to *L* and we have the *I–V* relation above the threshold:

(1) $$I_{D}=\frac{W}{L}\;C_{i}\mu \left(V_{G}-V_{\mathrm{th}}-\frac{V_{1}+V_{S}}{2}\right)\left(V_{1}-V_{S}\right)$$

Here, *V*_1_ would not exceed the saturation voltage *V*_D,SAT_, which is usually above *V*_D,SAT_ = *V*_G_ – *V*_th_ of conventional MOSFETs due to the transport in the various non‐gated semiconductors as discussed below.

In nongated semiconductors, the conduction mechanisms include: a) Fowler–Nordheim (F–N) tunneling via quantum mechanical tunneling or Poole–Frenkel (P–F) emission that occurs with rich structural defects;^[^ ^14^, ^15^ ^]^ b) Ohmic current or space charge limited current (SCLC) achieved by band‐conduction or thermally activated transport among localized states (e.g., at grain boundaries);^[^ ^16^ ^]^ c) ballistic transport without scattering (e.g., in nanotubes).^[^ ^17^ ^]^ Among them, Ohmic current and SCLC are mainly dominant at high current density in various semiconductors.^[^ ^18^, ^19^ ^]^ At the beginning of the non‐gated channel, the current density *J* depends on the local carrier density *n* and drift electric field *ε*:

(2) $$J\;=\left(n_{0}+n_{\mathrm{Sp}}\right)\;q\mu \varepsilon \;=\left(1+\eta \right)\;n_{0}q\mu \varepsilon$$

The subscript “0” and “Sp” refer to the free carriers at thermal equilibrium and the rest carriers forming space charge regions, respectively; *q* is the elementary charge; *η* is the space‐charge‐to‐equilibrium‐carrier ratio: *η*  = *n*_Sp_/*n*_0_  (Figure 1b). The *n*_Sp_ responsible for forming space charge regions will increase when increasing the dielectric relaxation time *τ*_R_ within which carriers are relaxed toward a uniform distribution, decreasing the carrier transit time *τ*_T_ within which carriers are swept out, or increasing the injected carrier density *n*_inj_. Thus, the average *η* is characterized by (see Note S1, Supporting Information for details):

(3) $$\eta \;=\frac{n_{\mathrm{Sp}}}{n_{0}}\;=\frac{n_{\mathrm{inj}}\tau_{R}}{n_{0}\tau_{T}}\;\approx \frac{WC_{i}\left(V_{G}-V_{\mathrm{th}}\right)V_{1}\varepsilon_{\mathrm{sc}}}{LSd{\left(qn_{0}\right)}^{2}}\frac{\mu_{1}}{\mu_{2}}$$

Here, *τ*_T_ ≈ *d* ^2^/(*μ*_2_Δ*V*) with Δ*V*  = *V*_2_  − *V*_1_, *τ*_R_ = *ε*_sc_/(*n*_0_ *qμ*_2_); *ε*_sc_, *d*, *S* are the permittivity, length, and current area of the nongated semiconductor; *V*_1_ is the potential at the end of the gated‐channel (with the upper limit *V*_G_ ‐ *V*_th_ or *V*_D_); *µ*_1_ and *µ*_2_ are the carrier mobility in the gated and nongated semiconductors.

The impacts of materials and device factors on *η* are summarized in Equation (3). The *η* is also an indicator for how severe the charges injected from the gated semiconductors are limited by the transport in the nongated semiconductors: a) with weak injection and fast relaxation (*η* ≪ 1), most injected carriers have been relaxed, giving uniform resistivity closed to Ohmic conduction (Figure 1c‐I); b) with strong injection and slow relaxation (*η* ≫ 1), most injected carriers kept unrelaxed, giving the non‐uniform resistivity closed to SCLC (Figure 1c‐III); c) with the intermediate degree of injection, the conduction is in the intermediate case (Figure 1c‐II); d) when the density of trap states *n*_t_ is comparable with that of mobile carriers, trap‐limited SCLC (or “trap‐fill limited,” TFL) dominates (Figure 1c‐IV). When estimating the average *η*, *V*_1_ ≈ (*V*_G_ − *V*_th_)/2 could be used. As *η* widely varies, a universal expression of the current density *J* is needed.

We simply assume *J*  =  *n*(*n*/*n*_t0_)^*δ* − 1^ *qμ*_0_ *ε*(*ε*/*ε*_t0_)^*κ* − 1^ =*An* ^*δ*^ *qμ*_0_ *ε* ^*κ*^, where *μ*_0_ is a characteristic mobility, *δ* − 1 and *κ* − 1 characterize the carrier‐density(*n*)‐dependent and field(*ε*)‐dependent mobility,^[^ ^13^, ^20^ ^]^ *n*_t0_ and *ε*_t0_ are the characteristic trap density and electric field, and *A* is a constant. In particular, *δ*  = *T*_c_/*T*  if density of trap states is in exponential distribution with energy and *T*_c_ is the characteristic temperature (*T*_c_ > *T*).^[^ ^21^, ^22^ ^]^ The general trap‐fill limited (TFL) SCLC for disordered semiconductors is (see Note S2, Supporting Information):

(4a) $$J_{\mathrm{SCLC}}=\;A^{\prime }\frac{\varepsilon^{\delta }}{q^{\delta -1}}\mu_{0}\frac{\Delta V^{\delta +\kappa }}{d^{2\delta +\kappa }}\propto \mu_{0}\frac{\Delta V^{\alpha }}{d^{\beta }}$$

where *A*′  =  *Aδ* ^*δ*^(2*δ* + *κ*)^*δ* + *κ*^/(*δ* + *κ*)^2*δ* + *κ*^, *α*  =  *δ* + *κ*, and *β*  =  2*δ* + *κ*. The power‐law *J–V* curves have been verified in many organic or compound semiconductor diodes.^[^ ^23^ ^]^ In on‐state transistors or diodes, the *n*‐dependence of *μ* is mainly considered as compared with *ε*‐dependence,^[^ ^19^, ^22^, ^24^ ^]^ therefore giving *κ* ≈ 1 and *β* ≈ 2*α* − 1. With few defects, *δ*  =  *κ*  =  1 and Equation (4a) returns to the classic SCLC (*α* = 2); with rich defects, the mobility is carrier‐density dependent: *μ*∝*n* ^*α* − 2^ *μ*_0_ (*α* > 2). In particular, *α*  = *T*_c_/*T*  + 1 when the density of defects per unit energy is exponentially distributed (with the width *T*_C_) in organic semiconductors.^[^ ^22^, ^24^ ^]^ For generality, Equation (4a) is rewritten into:

(4b) $$J\;=\left[Q_{0}{\left(\frac{\Delta V}{1}\right)}^{\alpha -1}{\left(\frac{1}{d}\right)}^{\beta -1}\right]\;\mu_{0}\;\frac{\Delta V}{d}=Q_{0}\;\mu_{0}\frac{\Delta V^{\alpha }}{d^{\beta }}$$

Here, *α* is referred as the charge transport factor and *Q*_0_ is referred as the charge density factor. The denominator 1 V and numerator 1 cm are introduced to keep the unit of *Q*_0_ as C cm^‐3^ (the same as *qn*) and so the term [*Q*_0_(Δ*V*/1)^*α* − 1^(1/*d*)^*β* − 1^] could be regarded as the equivalent charge density in the form of Ohmic law. A larger *Q*_0_ signifies the larger charge density if with the same *α*. Within a certain operational window, *α* and *Q*_0_ could be regarded as independent of the voltage drop Δ*V*. As shown in **Table** **1**, different conduction mechanisms could all be covered by Equation (4) (usually with *β*  =  2*α* − 1) as a general expression.

**Table 1:** Typical current density for nongated semiconductors.The mechanisms include Ohmic, SCLC, and some other cases (vacuum diode, emission, and tunneling). The values of *α*, *β*, *Q*_0_ are presented, where *K* and *V*_a_ are constants. The power factors *α* for emission and tunneling are derived in Note S3 (Supporting Information)

Conduction mechanism	Current density	*α*	*β*	*Q*_0_
Ohmic conduction	$J\;=n_{0}\;q\mu \frac{V}{d}$ (Ohm's law; when *η* ≪ 1)	1	1	*n*_0_ *q*
SCLC	$J\;=\frac{9}{8}\;\varepsilon_{\mathrm{sc}}\mu \frac{V^{2}}{d^{3}}$ ^[^ ^25^ ^]^ (Mott–Gurney law; when *η* ≫ 1)	2	3	$\frac{9}{8}\varepsilon_{\mathrm{sc}}$
Trap‐limited SCLC (n‐dependent µ)	$J\;=\;A^{\prime }\frac{\varepsilon_{\mathrm{sc}}^{\delta }}{q^{\delta -1}}\mu_{0}\frac{V^{\delta +1}}{d^{2\delta +1}}$ ^[^ ^22^ ^]^ [Equation 4; when *η* ≫ 1]	*δ* + 1 > 2	2*δ* + 1	$A^{\prime }\frac{\varepsilon_{\mathrm{sc}}^{\delta }}{q^{\delta -1}}$
Trap‐limited SCLC (n‐ and *ε*‐dependent‐µ)	$J\;=\;A^{\prime }\frac{\varepsilon_{\mathrm{sc}}^{\delta }}{q^{\delta -1}}\mu_{0}\frac{V^{\delta +\kappa }}{d^{2\delta +\kappa }}$ [Equation 4; when *η* ≫ 1]	*δ* + *κ* > 2	2*δ* + *κ*	$A^{\prime }\frac{\varepsilon_{\mathrm{sc}}^{\delta }}{q^{\delta -1}}$
Vacuum diode or ballistic SCLC	$J\;=\frac{4}{9}\;\varepsilon_{\mathrm{sc}}\sqrt{\frac{2q}{m}}\frac{V^{\frac{3}{2}}}{d^{2}}$ ^[^ ^18^, ^25^ ^]^ (Child–Langmuir law)	1.5	2	$\frac{4}{9}\varepsilon_{\mathrm{sc}}\sqrt{\frac{2q}{m}}$
Emission (P–F and etc.)	$J∼V\exp(K\sqrt{V})$ ^[^ ^25^ ^]^	≈2	–	–
Tunneling (F–N and etc.)	$J∼V^{2}\exp\left(-V_{a}/V\right)$ ^[^ ^25^ ^]^	≈2	–	–

## 3 The Device Performance

### 3.1 Current–Voltage Relations

How materials and device factors affect conduction mechanisms could be understood by combining Equations. (3) and (4). For example, the transport factor *α* is very sensitive to *n*_0_ as $\eta \;\approx 1/n_{0}^{2}$ and, thus, if the semiconductor become rich in carrier‐generating states (*η* ≪ 1), the SCLC‐to‐Ohmic transition could occur (*α* decreases from 2 toward 1). Conversely, if the injection from the gated channel (*C*_i_ *V*_G_) significantly increases or the nongated channel becomes rich in trapping states (*η* ≫ 1), the Ohmic‐to‐SCLC transition may occur or even to the trap‐limited SCLC (*α* increases from 1 toward 2 and beyond).

Taking a drain‐offset transistor, or similarly a transistor‐diode hybrid integration, as an example (Figure 1b with *V*_2_ *= V*_D_), the gated and nongated channel (Equations 1 and 4) are coupled and mutually limit each other:

(5) $$\begin{array}{ccc} I_{D} & = & \frac{W}{L}\;\mu_{1}C_{i}\left(V_{G}-V_{\mathrm{th}}-\frac{V_{1}+V_{S}}{2}\right)\;\left(V_{1}-V_{S}\right)\\ & = & Q_{0}\;S\mu_{2}\frac{{\left(V_{D}-V_{0}-V_{1}\right)}^{\alpha }}{d^{\beta }} \end{array}$$

Here, *C*_i_ = *ε*_ox_/*t*_ox_ with *ε*_ox_ as permittivity and *t*_ox_ as the thickness of the insulating layer below or above the gated semiconductor, and *V*_0_ is the onset voltage for the nongated channel (due to injection barriers or trap states). In the following, *V*_0_ is omitted for simplicity and could be included by replacing *V*_D_ with *V*_D_‐*V*_0_ whenever needed. For a drain‐offset transistor, *S*  =  *Wt*_sc_ with *t*_sc_ as the thickness of the nongated semiconductor. By defining the partial voltage coefficient *γ*  = (*LSQ*_0_ *μ*_2_)/(*Wd* ^*β*^ *C_i_μ*_1_)  and assuming a small contact effect (*V*_S_ ≪ *V*_1_), Equation (5) becomes its voltage form:

(6) $$\left(V_{G}-V_{\mathrm{th}}-\frac{V_{1}}{2}\right)\;V_{1}=\;\gamma {\left(V_{D}-V_{1}\right)}^{\alpha }$$

As *V*_1_ < *V*_D_, the second‐order Taylor series is applied to the right and the accurate and approximated solutions are:

(7) $$\begin{array}{ccc} V_{1} & = & \left(-b-\sqrt{b^{2}-4ac}\right)/\left(2a\right)\\ & \approx & \left(V_{G}-V_{\mathrm{th}}\right)-\sqrt{{\left(V_{G}-V_{\mathrm{th}}\right)}^{2}-2\gamma V_{D}^{\alpha }} \end{array}$$

where $a\;=\;1+\gamma \alpha \left(\alpha -1\right)V_{D}^{\alpha -2}$, $b\;=\;-2(V_{G}-V_{\mathrm{th}}+\alpha \gamma V_{D}^{\alpha -1})$, and $c\;=\;2\gamma V_{D}^{\alpha }$ (Note S4, Supporting Information). The accurate solution is used below and the approximated solution is for very small *γ* (e.g., when the nongated channel is with low conductance).

**Figure 2:**
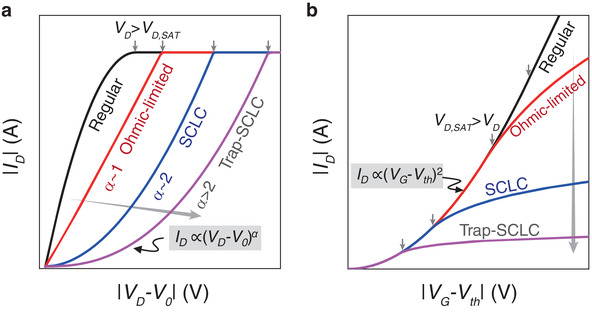
Impacts of transport on *I*–*V* characteristics (qualitative). a) Output and b) transfer characteristics according to Equation (5). The trap‐limited (or TFL) SCLC is denoted as “Trap‐SCLC” (the same below). The small arrows indicate the positions where *V*_D_ exceeds *V*_D,SAT_ in (a) or *V*_D,SAT_ exceeds *V*_D_ in (b). The gray arrows point to the direction in which *η* increases and that the device current is increasingly limited by the nongated semiconductors.

The *I*–*V* relations described by Equations (5 and 7) with *V*_S_ ≈ 0 are qualitatively illustrated in **Figure** **2**. In output characteristics (Figure 2a), |*I*_D_| increases with |*V_D_* ‐ *V*_1_ ‐ *V*_0_|*^*α*^* (approximately with |*V*_D_ ‐ *V*_0_|^*α*^ as often |*V*_1_| ≪ |*V*_D_|) until *V*_D_ reaches the saturated voltage *V*_D,SAT_, beyond which the current saturates due to pinch‐off. In transfer characteristics (Figure 2b), |*I*_D_| increases with (*V*_G_ ‐ *V*_th_)^2^ until |*V*_D,SAT_|>|*V*_D_|, beyond which the current is limited by the nongated channel. When the nongated channel has high conductance (large *γ*), the device behaves like a regular transistor; otherwise, the current is limited by the nongated channels (small *γ*) in the case of Ohmic current, SCLC, or trap‐limited SCLC. Conduction mechanisms could change if changing materials or device factors according to Equation (3).

**Figure 3:**
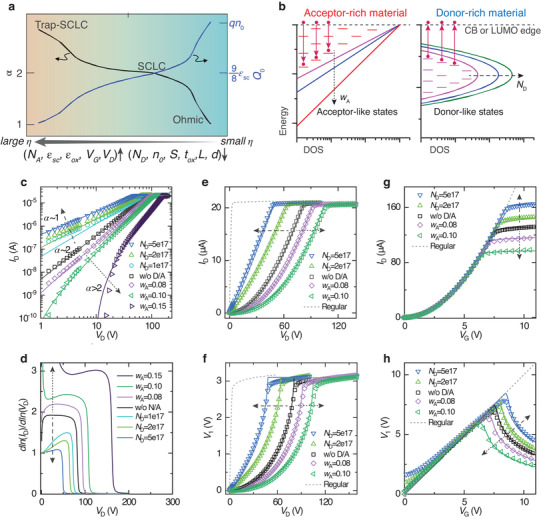
Impacts of materials on *I*–*V* characteristics (quantitative). a) A summary of the impacts of materials and device factors on the indicator *η*, the transport factor *α*, and charge density factor *Q*_0_. The grey arrow indicates the direction that *η* increases, if *N*_A_, *ε*_sc_, *ε*_ox_, *V*_G_, or *V*_D_ increases and *N*_D_, *n*_0_, *S, t*_ox_, *L*, or *d* decreases. Here, *N*_A_ and *N*_D_ represent the characteristic density of donor‐ and acceptor‐like states. b) Schemes of density of states (DOS) of acceptor‐rich material and donor‐rich material. c) *I*_D_–*V*_D_ curves in the semilog scale. d) Extracted *α*  =  ∂ln*I*_D_/∂ln*V*_D_. e,f) *I*_D_–*V*_D_ curves in the linear scale (*V*_G_ = 3 V) and the corresponding *V*_1_ read from a point near the end of the gated channel (7.95 out of 8 µm), which gets almost saturated when *V*_D_ > *V*_D,SAT_. g,h) *I*_D_–*V*_G_ curves (*V*_D_ = 200 V) and the corresponding *V*_1_, which starts decaying when *V*_D,SAT_ > *V*_D_. The TCAD simulated data are drawn in dots and fitting by using Equations 5 and 7 are drawn in curves. In (d–h), results of a regular TFT are shown for references. The dotted (or dashed) arrows represent the direction that acceptor‐like (or donor‐like) states increase.

The impacts of material and device factors on the ratio *η*, charge transport factor *α*, and charge density factor *Q*_0_ are summarized in **Figure** **3**a. The original data are given in Figure S1 (Supporting Information). As an example, the impacts of donor‐like and acceptor‐like states on *I–V* characteristics are shown in Figure 3b–h, including the 2D‐TCAD simulation (dots) and the fitting using Equations. (5 and 7) (curves). The 2D‐TCAD simulations were performed by solving Poisson equations and continuity formulas in fine grids and parameters are shown in Table S1 (Supporting Information). For donor‐like states, the peak energy is fixed below the conduction band edge (CB, or lowest unoccupied molecular orbit LUMO) with the same characteristic width (*w*_D_), while the maximum number of the Gaussian‐distribution (*N*_D_) is varied (Figure 3b). For acceptor‐like states, the maximum of the exponentially distributed acceptor‐like tail states (*N*_A_) is fixed as 10^18^ cm^–3^, while the characteristic width (*w*_A_) is varied (Figure 3b). With more donor‐like states, e.g., increasing *n*_0_ = 10^15^ cm^–3^ to *n*_0_ = 10^16^ cm^–3^, the estimated ratio *η* by Equation (3) sharply decreases from 151 to 1.5 (using *V*_1_ = *V*_G_/2 = 1.5 V), suggesting that *α* would change from 2 toward 1. Consistently, the transport factor *α* decreases from 2 to 1 as *N*_D_ increases, as shown in Figure 3c,d. By contrary, *α* increases beyond 2 as the DOS of acceptor‐like states broadens (*w*_A_ increases). The evolution of output and transfer characteristics (Figure 3e–h) could all be understood by that: when *N*_D_ increases, *Q*_0_ (and thus *γ*) increases, leading to the increased *V*_1_ (and thus *I*_D_) and decreased *V*_D,SAT_; the opposite occurs when *w*_A_ increases. Using Equation (5) with a single set of *Q*_0_ and *α* provides good fitting to both *I*_D_ and *V*_1_ simultaneously for all the cases (Figure 3e–h), validating the simplified physical picture.

### 3.2 Potential and Field Evolution

**Figure 4:**
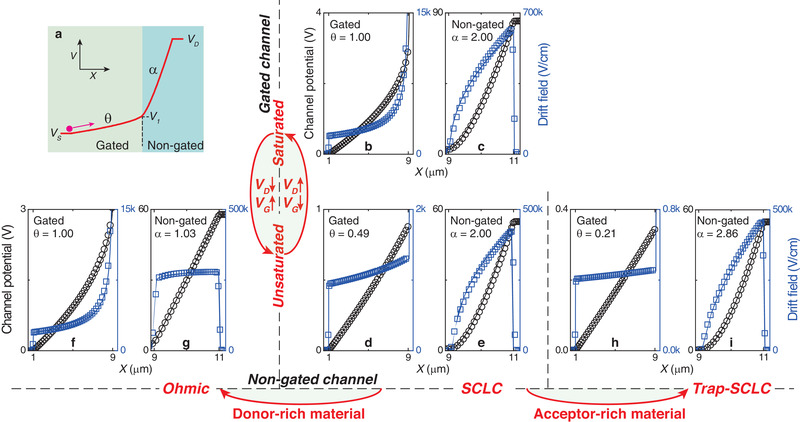
Impacts of materials on channel potential (black) and drift field (blue). a) A scheme of potential distribution. The gated channel is from *X* = 1 to 9 µm, the nongated channel is from *X* = 9 to 11 µm, and *V*_G_ = 3 V. The source potential *V*_S_ is referred to as zero. The gated channel changes from the saturated (b,c, *V*_D_ = 85 V) to unsaturated (d,e, *V*_D_ = 55 V). With *V*_D_ = 55 V, the nongated channel changes from the SCLC (d,e, intrinsic semiconductor) to Ohmic conduction in donor‐rich materials (f,g, with *N*_D_ = 5 ×  10^17^ cm^–3^ eV^–1^ and *w*_D_ = 0.1 eV), or to trap‐limited SCLC in acceptor‐rich materials (h,i, with *N*_A_ = 10^18^ cm^–3^ eV^–1^ and *w*_A_ = 0.1 eV). Curves are the calculation by Equation (8) and well fit the TCAD simulation (dots). The saturation degree *θ* (from calculated) characterizing the gated channels and the charge transport factor *α* (for fitting) characterizing the non‐gated channels are shown.

The evolution of local channel potential *V*(*x*) can be derived from Equation. (5):

(8a) $$V\;\left(x\right)=\left(V_{G}-V_{\mathrm{th}}\right)\;\left(1-\sqrt{1-\theta \frac{x}{L}}\right),\;\text{for}\;0<x<L$$

(8b) $$V\;\left(x\right)=V_{1}\;+\Delta V{\left(\frac{x-\;L}{d}\right)}^{\beta /\alpha },\;\text{for}\;L\leq x\leq L+d$$

where *θ* is defined as the saturation degree, *θ*  = 2*V*_1_(*V*_G_ − *V*_th_ − *V*_1_/2)/(*V*_G_ − *V*_th_)^2^ , and 0 < *θ* ≤ 1. It quantifies the impacts of drain and gate voltages on the operational regime: when *θ* ≪ 1 or *θ* = 1, the gated channel is in the linear or saturated regime, respectively. In general, distributions of *V*(*x*) are characterized by *θ* and *α* (illustrated in **Figure** **4**a) and will change when voltages or material properties significantly change.

The impacts of voltage scanning are exemplified in Figure 4b,c for a saturated, gated channel and in Figure 4d,e for an unsaturated, gated channel with the reasons for the transition (i.e., changing *V*_D_ or *V*_G_). The impacts of materials are exemplified in Figure 4f,g by inducing donor‐like states with Ohmic conduction (*α* ≈ 1) in the nongated channel and in Figure 4h,i by inducing acceptor‐like states with trap‐limited SCLC (*α* >2), respectively. At the meantime, the gated channel also changes to the saturated regime (Figure 4f, *θ*  =  1) or the linear regime (Figure 4h, *θ* ≪ 1) due to the increased or decreased partial voltage coefficient *γ* (and thus *V*_1_), respectively. For all the cases, the calculations by Equation (8) (curves) agree well with the 2D TCAD simulation (dots). Also, we could obtain the drift electric field by *E_X_* (*x*) =   − d*V*/d*x*, Joule heating power density by *p* (*x*) = |*JE_X_* | , velocity of carriers (including hot carriers) by *v_X_* (*x*) =  *μE_X_*, and carrier density by *n* (*x*) = *J*/(*qμE_X_*)  by using Equations (5 and 8) or probing *V*(*x*) in experiments for partially gated or even regular transistors.

### 3.3 Current Saturation

Current saturation occurs when carrier concentration becomes depleted near the end of the gated channel, with *V*_1_ reaching (*V*_G_ *‐ V*_th_) and *V*_D_ reaching *V*_D,SAT_. As *V*_D,SAT_ is the turning point in *I–V* curves (Figure 2) and critical for stabilizing current and tolerating signal fluctuations, it is derived from Equation (7):

(9) $$\begin{array}{ccc} V_{D,\mathrm{SAT}} & = & \left(V_{G}-V_{\mathrm{th}}\right)\;+\sqrt[{\alpha }]{{\left(V_{G}-V_{\mathrm{th}}\right)}^{2}/\left(2\gamma \right)}\\ & \approx & \sqrt[{\alpha }]{\frac{Wd^{\beta }}{2LS}\frac{\varepsilon_{\mathrm{ox}}}{t_{\mathrm{ox}}Q_{0}}\frac{\mu_{1}}{\mu_{2}}{\left(V_{G}-V_{\mathrm{th}}\right)}^{2}} \end{array}$$

**Figure 5:**
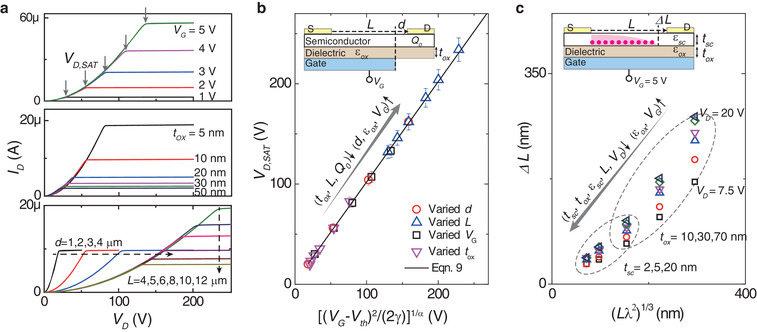
Impacts of materials and device factors on current saturation. a) For drain‐offset transistors, output curves with various *V*_G_ (top), *t*_ox_ (middle), *L* and *d* (bottom). *V*_D,SAT_ is illustrated by arrows. b) The extracted *V*_D,SAT_ (dots) are compared with the calculated results from Equation (9) with *α* = 2 (the line). The arrow indicates that *V*_D,SAT_ will increase if *t*_ox_, *L*, or *Q*_0_ decreases and *d*, *V*_G_, or *ε*_ox_ increases. c) For regular transistors, extracted Δ*L* of devices with various *λ* by changing *t*_ox_ or *t*_sc_ (dashed ovals). *ΔL* is the distance from the drain to the pinch‐off point, where the interfacial potential reaches *V*_G_ – *V*_th_. Here, *V*_D_ ranges from 7.5 to 20 V with the step of 2.5 V. The arrow indicates that *ΔL* will decrease if *t*_sc_, *t*_ox_, *ε*_sc_, *L* or *V*_D_ decreases and *ε*_ox_ or *V*_G_ increases.

Here, *ε*_ox_ and *t*_ox_ are the permittivity and thickness of the dielectric layer and *S* = *Wt*_sc_. The approximate equal sign holds when *γ* is small or, instead, *V*_D,SAT_ approaches the classic (*V*_G_ *‐ V*_th_) when *γ* is large. Accordingly, *V*_D,SAT_ increases linearly with (*V*_G_ *‐ V*_th_)^2^ or (*V*_G_ *‐ V*_th_) with ideal Ohmic conduction or SCLC, respectively. We use a trap‐free semiconductor in simulations to examine *V*_D,SAT_ of devices with varied parameters (*V*_D_, *t*_ox_, *d*, and *L*, **Figure** **5**a), which fall in the same line calculated by Equation (9) with *α* = 2 (SCLC regime, Figure 5b and Figure S2, Supporting Information). The materials or device factors that lead to increased *V*_D,SAT_ are also indicated by the gray arrow.

Interestingly, the method helps to answer another long‐standing problem: how to estimate the drain depletion‐region length (Δ*L*, Figure 5c) in a saturated TFT? As this region is depleted (*n*_inj_/*n*_0_ ≫ 1) and narrow (*τ*_R_/*τ*_T_ ≫ 1), Δ*L* could be estimated by using Equation (5) with *V*_1_ = *V*_G_ ‐ *V*_th_, *V*_2_ = *V*_D_, *d* = Δ*L* and the SCLC limit:

(10) $$\begin{array}{ccc} \Delta L & = & \sqrt[{\beta }]{\frac{2LQ_{0}t_{\mathrm{sc}}}{C_{i}}\frac{\mu_{2}}{\mu_{1}}\frac{{\left(V_{D}-V_{G}+V_{\mathrm{th}}\right)}^{\alpha }}{{\left(V_{G}-V_{\mathrm{th}}\right)}^{2}}}\\ & \approx & \sqrt[{3}]{\frac{9}{4}{\left(\frac{V_{D}}{V_{G}-V_{\mathrm{th}}}-1\right)}^{2}}\cdot \sqrt[{3}]{L\lambda^{2}} \end{array}$$

where $\lambda \;=\sqrt{\varepsilon_{\mathrm{sc}}t_{\mathrm{sc}}/C_{i}}\;$ is the natural length.^[^ ^26^ ^]^ The Δ*L* in accumulation‐mode, thin‐film based transistors is predicted to increase with *Lλ* ^2^, different from conventional inversion‐mode, pn‐junction based MOSFETs.^[^ ^27^ ^]^ This is consistent with the extracted Δ*L* by varying *t*_sc_ or *t*_ox_ in TCAD simulation (Figure 5c and Figure S3, Supporting Information). The undesired channel length modulation^[^ ^27^ ^]^ characterized by *L*/(*L* − Δ*L*) would be intensified in short‐channel TFTs, but could be weakened by decreasing *λ* (Note S5, Supporting Information). It also quantifies the advantages of using ultra‐thin semiconducting films in short‐channel FETs in terms of weakening the channel modulation.

## 4 Various Applications

### 4.1 Applications with Various Semiconductors

When using organic semiconductors in transistors or transistor–diode integrations,^[^ ^28^ ^]^ charge carriers are generally intrinsically low (*n*_0_ < 10^18^ cm^–3^) and thus usually with *η* ≫ 1 and *α* ≈ 2. But charge transport of polarons could be limited by structural disorders so that it takes the forms of trap‐and‐release, variable range hopping, charge transfer, nuclei tunneling, and etc.^[^ ^20^ ^]^ The macroscopic conductance as a function of temperature and gate‐field could be described by the thermal activation of carriers near the exponentially distributed tail states.^[^ ^22^, ^24^ ^]^ In these cases, *α*  = *T*_c_/*T*  + 1 > 2 is expected, especially in a disordered film with localized tail states or trapping states.^[^ ^19^, ^24^, ^29^ ^]^ We have shown that various transport mechanisms of organic semiconductors could be universally described by the generalized Einstein relation,^[^ ^20^, ^30^ ^]^ so that the gate‐ and temperature‐dependent mobility of organic semiconductors could be included in the current framework. In addition, organic FETs usually suffer from significant contact injection barriers that lower the on‐current and cut‐off frequencies^[^ ^15^, ^31^ ^]^ and, thus, the contact potential *V*_S_ should be considered.

When using metal‐oxide semiconductors as the nongated channels, the carrier density and field‐dependent transport mechanisms include the trap‐limited conduction, variable range hopping, and percolation.^[^ ^32^ ^]^ As the intrinsic carrier concentration is also usually low with abundant defects,^[^ ^33^ ^]^ *η* ≫ 1, *α* > 2, and a small value of *Q*_0_ close to or below the SCLC limit would be expected. In contrast, when using semiconductors with high carrier densities as the nongated channels for light‐emitting or detecting, e.g., perovskites and 2D narrow‐bandgap materials, we expect *η* ≪ 1, *α*  ≈  1, and *Q*_0_ ≈ *qn*_0_. The mentioned materials will be studied in various devices in the following section.

### 4.2 Applications in Various Transistors and Hybrid Integration

**Figure 6:**
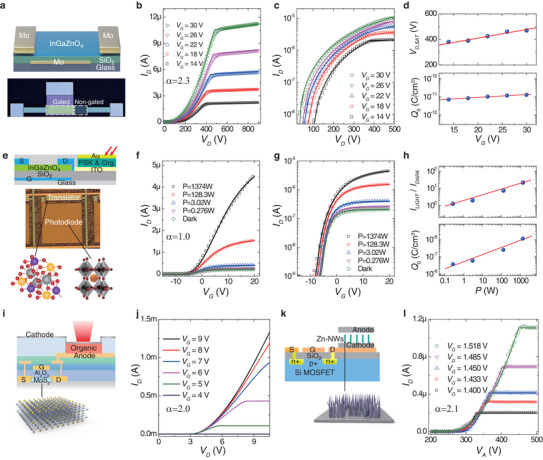
Various transistors and device integrations. a) A schematic representation and an optical picture of an drain‐offset TFT. b,c) Output characteristics in the linear scale or semi‐log scale. The dots are experimental data and the curves are the fitting results by using Equations (5 and 7). d) Values of extracted *V*_D,SAT_ (top) and *Q*_0_ (bottom) are shown as a function of *V*_G_. e) A schematic representation and an optical picture of the transistor–photodiode hybrid integration. The perovskite‐organic layers are denoted as “PSK & Org.” f,g) Transfer characteristics (*V*_D_ = 0.1 V) in the linear or semi‐log scale. h) Light‐to‐dark current ratio (top) and the charge density factor *Q*_0_ for the fitting (bottom) as a function of illumination power. i) A schematic representation of the MoS_2_ transistor integrated with OLED. j) Calculated output characteristics using the parameters in the reference^[^ ^38^ ^]^ with the ideal SCLC, which agrees well with the reported data.^[^ ^28^ ^]^ k) A schematic representation of the Si‐FET and ZnO–nanowire–emitter hybrid integration. l) Field‐emission *I–V*_A_ curves. The dots are experimental data and the curves are the calculation or fitting by using Equations (5 and 7).

As the first example, transistors in the drain‐offset structure have been used to stabilize current^[^ ^2^ ^]^ or to make high‐voltage TFTs^[^ ^34^ ^]^ for driving field‐emitters, piezoelectric actuators, or integrated MEMS.^[^ ^35^, ^36^ ^]^ Drain‐offset transistors were fabricated based on amorphous N_2_‐doped InGaZnO_4_ semiconductor (*t*_sc_ = 60 nm), which is chosen for its relatively large bandgap (≈3 eV) and low intrinsic carrier concentrations^[^ ^37^ ^]^ to investigate the current limitation from the nongated channels. The methods of fabrications and measurements are the same as described elsewhere.^[^ ^35^ ^]^ The electrodes are Mo (*L* = 200 µm, *d* = 100 µm, and *W* = 40 µm) and the dielectric layer is SiO_2_ (*t*_ox_ = 300 nm). The measured output characteristics are shown in **Figure** **6**b,c, with the dots and curves representing the experimental data and fitting, respectively. The estimated *η* as an indicator is about 192 due to low carrier density in the N_2_‐doped InGaZnO_4_ (*n*_0_ ≈ 10^15^ cm^–3^, *V*_G_ = 30 V). The fitting results are calculated by the total resistance *R*_tot_ with the channel resistance (*R*_CH_) and a back‐channel resistance (*R*_BACK_) in parallel. *R*_CH_ is calculated by Equations (5 and 7) using the same parameter *α* (with *β* = 2*α*‐1) and a fitting parameter *Q*_0_, while *R*_BACK_ is extracted by *R*_BACK_ = ∂*V*_D_/∂*I*_D_  beyond *V*_D,SAT_. The simple fittings agree well with the experimental data with *α* = 2.3 (trap‐limited SCLC), corresponding well with the disordered transport in amorphous InGaZnO_4_. The values of *V*_D,SAT_ increase almost linearly with the *V*_G_ (Figure 6, top) and this is also consistent with the above theories. The charge density factor *Q*_0_ changes slightly when varying *V*_G_ (Figure 6, bottom). Thus, the drain‐offset, high‐voltage TFTs could be understood by the above physical framework.

Transistor hybrid integration, e.g., with photo‐diodes, light‐emitting diodes, field emitters, or others for various functions, could also be effectively simplified by the above approach. As the first example, the transistor‐photodiode integration was fabricated by integrating a vacuum‐deposited InGaZnO_4_ TFT and solution‐processed a vertical photodiode based on perovskite‐organics [ITO‐SnO_2_‐(FASnI_3_)_0.6_(MAPbI_3_)_0.4_‐poly(3‐hexylthiophene)‐Au, Figure 6e]. The perovskite semiconductor is chosen as a representative, light‐sensitive semiconductor with relatively high carrier concentrations and Ohmic conduction in a diode structure.^[^ ^39^, ^40^ ^]^ The methods of fabrication and measurement are the same as described elsewhere.^[^ ^39^ ^]^ The parameters for experimental devices are: *W* = 1600 µm, *L* = 40 µm, *d* = 0.5 µm, *t*_sc_ = 60 nm, *t*_ox_ = 300 nm (SiO_2_), and *V*_d_ = 0.1 V. Due to the high carrier density in perovskite semiconductors, the photodiodes exhibit Ohmic conduction behaviors in *J*–*V* characteristics^[^ ^39^ ^]^ so that *η* ≪ 1 and *α* = 1 is used here. The transfer curves are shown in the linear or semilog scale with the fitting curves (Figure 6f,g). The mobility parameters are set the same for all the curves with different illuminations, whereas fitting parameters *Q*_0_ are varied for each illumination power and show the same trend with the light‐to‐dark current ratio (Figure 6h). These results are consistent with the above discussions and verify that *Q*_0_ reflects the photogenerated carrier density in the non‐gated channels (photodiodes). As the second example, the performance of MoS_2_‐TFT and OLED integration (Figure 6i) is predicted by using Equations (5 and 7) with the device and material parameters as reported in reference^[^ ^38^ ^]^ and with the ideal SCLC, giving the results (*α* = 2, Figure 6j) close to the experimental data reported in reference.^[^ ^38^ ^]^ The 2D semiconductors are the extensively studied candidates for ultra‐thin, fast logic devices.^[^ ^10^ ^]^ As the third example, Si‐FET and ZnO–nanowire–emitter integration (Figure 6k) were fabricated as described elsewhere^[^ ^41^ ^]^ and the *I–V* characteristics are shown in Figure 6l. The nanowire emitter is studied here as it could be used in the source of fast electron beams for microscope or nanolithography technologies.^[^ ^42^ ^]^ The *I*–*V* characteristics could be fitted by using Equations (5 and 7) with *α* = 2.1, supporting that the field‐emission by F–N tunneling into vacuum at large anode bias *V*_A_ could also be approximated by Equation (5). These results confirm the proposed physical framework could also be applied to understand and simplify transistor hybrid integration.

### 4.3 Applications in Fast Computing of Complex Transistors

**Figure 7:**
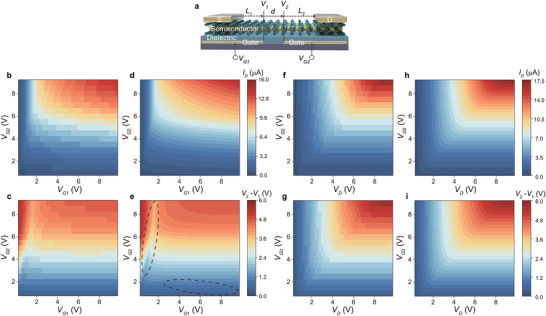
Exemplary results of fast computing split‐gate transistors. a) A schematic representation of the transistor structure. For transfer characteristics, b,d) the current *I*_D_ and c,e) the potential drop *V*_2_–*V*_1_ at varied *V*_G1_ and *V*_G2_ with fixed *V*_D_ = 6 V, where (b, c) is obtained from the TCAD simulation (0.5 V step) and (d, e) are from calculations by Equation (11) (0.05 V step). The computing time on the same computer is about 50 min and 2 s, respectively. For output scanning, the data for varied *V*_D_ and *V*_G2_ with fixed *V*_G1_ = 6 V are shown in (f–i) in the same order.

The above theory provides opportunities for fast computing or design aid of complex transistors. As a demonstration, a compiled HTML file is provided to calculate drain‐offset transistors, mid‐gate transistors, solid‐state vacuum triodes, split‐gate transistors, and transistor hybrid integration according to the input parameters (see Figures S4 and S5, Supporting Information). The calculated results presented in this manuscript could be obtained by using this file. As an example, split‐gate transistors have been made (e.g., with MoS_2_ or WSe_2_)^[^ ^8^, ^43^ ^]^ for fast sensing and computing applications and could be regarded as the gated/nongated/gated structure. A typical device structure is illustrated in **Figure** **7**a. Denote the gate voltages as *V*_G1_ (near the source) and *V*_G2_ (near the drain) and the current is:

(11) $$\begin{array}{ccc} I_{D} & = & \frac{W_{1}}{L_{1}}\;\mu_{1}C_{i}\left(V_{G1}-V_{\mathrm{th}1}-\frac{V_{1}+V_{S}}{2}\right)\;\left(V_{1}-V_{S}\right)\\ & = & SQ_{0}\mu_{2}\;\frac{{\left(V_{2}-V_{1}\right)}^{\alpha }}{d^{\beta }}\\ & = & \frac{W_{3}}{L_{3}}\;\mu_{3}C_{i}\left(V_{G2}-V_{\mathrm{th}2}-\frac{V_{D}+V_{2}}{2}\right)\left(V_{D}-V_{2}\right) \end{array}$$

here, *V*_1_ and *V*_2_ are the potential at the end and beginning of the two gated channels. Calculation methods are given in Note S6 (Supporting Information). Exemplary results of calculated transfer characteristics with the same set of parameters are presented by plotting the contours of *I*_D_ and the voltage drop across the non‐gated channel *V*_2_ ‐ *V*_1_ in Figure 7d,e, which agree well with the 2D‐TCAD simulation shown in Figure 7b,c but consume about 1000 times less time. In particular, the asymmetric impacts of *V*_G1_ and *V*_G2_ on *I_D_* and *V*_2_–*V*_1_ are clearly observed with details in Figure 7d,e, dashed ovals. Output characteristics are shown in Figure 7e–h. The results demonstrate that the simplified physical picture may provide a physical‐meaningful platform for few‐shot learning to train parameters and to model and predict the performance of complex transistors.

For computing other transistors or transistor integration in Figure 1a, similar approaches could be applied by modifying Equation (5). For example, mid‐gate transistors and solid‐state vacuum triodes (or so‐called “static induction transistors”)^[^ ^3^, ^44^ ^]^ (Figure 1a) could be treated by rewriting Equation (5) corresponding to the nongated/gated/nongated structure (Note S7, Supporting Information). In general, mid‐gate transistors usually have Ohmic conduction in the non‐gated channels to allow Ohmic injection (e.g., Ga_2_O_3_ transistors^[^ ^45^ ^]^), while static induction transistors usually have SCLC in the non‐gated channels to keep the low off‐current.

## 5 Conclusion

The charge transport of semiconductors in transistors with nontrivial gates or hybrid integration could be simplified and understood in the same physical picture of “charge carriers from gated into nongated semiconductors.” The conduction in the nongated semiconductors is a synergetic result of charge transport in the gated semiconductors and carrier relaxation, transit, and transport in the non‐gated semiconductors. A general theoretical framework is derived to describe *I–V* relation, current saturation, evolution of potential and drift field for various transistors with nontrivial gates or transistor–diode integration. Within the framework, how materials and device factors determine the performance could be briefly understood by: a) the space‐charge‐to‐free‐carrier ratio *η* as an indicator that characterizes how severely the charges injected from the gated semiconductor are limited by the transport in the nongated semiconductor; b) the charge transport factor *α* and charge density factor *Q*_0_ that characterize the conduction mechanisms from Ohmic to SCLC and then to trap‐limited SCLC; c) the partial voltage coefficient *γ* that characterizes the voltage distribution between the coupled channels (an increased *γ* leads to an increased *V*_1_ across the gated channel). The understanding has been verified by numerical simulations and agrees well with device experiments and a device calculator is demonstrated. Having general applicability, the approach may be combined with specific properties of semiconductors and devices and provides a straightforward way to quickly understand, model, design, and analyze complex transistors or hybrid integrations and the semiconductors in them.

## 6 Experimental Section

### Device Simulation

The 2D‐TCAD simulations were performed by solving Poisson equations and continuity formula in fine grids. The device parameters are *W =* 1000 µm, *L* = 8 µm, *d* = 2 µm, *t*_sc_ = 20 nm*, µ*_1_ *= µ*_2_ *=* 1 cm^2^ V^‐1^ s^‐1^, and *C*_i_ *=* 11 nF cm^–2^. More details of simulation and fitting parameters are given in Tables S1–S3 and Notes S5and S6 in Supporting Information.

### Device Fabrication

Drain‐offset transistors were fabricated based on amorphous N_2_‐doped InGaZnO_4_ semiconductor (*t*_sc_ = 60 nm). The methods of fabrications and measurements are similar with our previous studies.^[^ ^35^ ^]^ Bottom‐gate, inverted staggered TFTs with an offset‐gate were fabricated on glass substrates. The electrodes are Mo (*L* = 200 µm, *d* = 100 µm, and *W* = 40 µm) and the dielectric layer is SiO_2_ (*t*_ox_ = 300 nm). Gate electrodes were formed by the deposition of a 200 nm thick Mo layer that was subsequently patterned by wet etching. The gate insulator was then deposited by plasma‐enhanced chemical vapor deposition (PECVD). A 60 nm thick InGaZnO_4_ film was sputtered on the gate insulator and etched to form an active layer. The InGaZnO_4_ deposition was conducted under nitrogen gas to introduce nitrogen doping, which helped to improve the stability of InGaZnO_4_. In sputtering, the gas flow was Ar/O_2_/N_2_ = 30:0.5:1 in the unit of sccm with the power of 900 W. The source and drain electrodes were formed by sputtering and lift‐off processes. A passivation layer was deposited by PECVD and electrode holes were etched by reactive ion etching. Finally, the devices were annealed at 350 °C for 1 h in nitrogen atmosphere.

Transistor–photodiode integration was fabricated by integrating a vacuum‐deposited InGaZnO_4_ TFT and solution‐processed a vertical photodiode based on perovskite‐organics [ITO‐SnO_2_‐(FASnI_3_)_0.6_(MAPbI_3_)_0.4_‐poly(3‐hexylthiophene)‐Au]. The methods of synthesis of (FASnI_3_)_0.6_(MAPbI_3_)_0.4_, fabrication of photodiodes, and integration of photodiodes with TFTs are the same as described elsewhere.^[^ ^39^ ^]^ The parameters for experimental devices are: *W* = 1600 µm, *L* = 40 µm, *d* = 0.5 µm, *t*_sc_ = 60 nm, *t*_ox_ = 300 nm (SiO_2_), and *V*_d_ = 0.1 V. The area *S* for a pixel of photodetector is 1925 µm × 1925 µm.

Si‐FET/ZnO–nanowire–emitter integration was fabricated by growing ZnO nanowires on the drain electrode of a MOSFET, as described elsewhere.^[^ ^41^ ^]^ N‐channel enhancement MOSFET (Infineon: BSP 324) was used for the high maximum‐rating drain–source voltage (400 V). The layers of Cr (200 nm) and Zn seed material (100 nm) were selectively deposited on the drain electrode of the MOSFET by sputtering. A solution was prepared by mixing zinc nitrate hexahydrate and hexamethylenetetramine (1:1) with the concentration of Zn^2+^ of 2 µmol L^‐1^. Then the substrates were suspended with top‐side down in the solution at 80 °C for 18 h for growing ZnO nanowires.

### Device Characterization and Analysis

Drain‐offset transistors were measured for high‐voltage test by using a high‐voltage semiconductor test system (Keithley 2657A and Keithley 2450). In the drain‐offset InGaZnO_4_ transistors (Figure 6), the common parameters for fitting are: *µ*_1_ *=* 10 cm^2^ V^‐1^ s^‐1^, *µ*_2_ *=* 1 cm^2^ V^‐1^ s^‐1^, *V*_th_ = 0.75 V, and the charge transport factor *α* = 2.34. The charge density factor *Q*_0_ depending on *V*_G_ are *Q*_0_ = 1.3  ×  10^–11^, 1.1  ×  10^–11^, 9.9 ×  10^–12^, 8.2  ×  10^–12^, or 6.9  ×  10^–12^ C cm^3^, for *V*_G_ = 30, 26, 22, 18, or 14 V.

For transistor–photodiode integration with a InGaZnO_4_‐TFT and a PSK‐photodiode, the electrical characteristics were characterized in air by a semiconductor parameter analyzer (Agilent, B1500A) in the dark or under illumination with a LED (wavelength 850 nm). The common parameters for fitting are: *µ*_1_ = 8[1 − exp ( − (*V*_Gt_/17)^2^)] cm^2^ V^‐1^ s^‐1^ *, µ*_2_ = 0.1 cm^2^ V^‐1^ s^‐1^, *α* = 1. The charge density factor *Q*_0_ and threshold voltage *V*_th_ depending on illumination power are *Q*_0_ = 11.1 ×  10^–7^, 2.4 ×  10^–7^, 5.7  ×  10^–8^, 3.6  ×  10^–8^, and 2.9 ×  10^–8^ C cm^‐3^ and *V*_th_ = ‐8.5, ‐7.8, ‐7.0, ‐6.8, or ‐6.5 V for *P* = 1374, 128.3, 3.02, 0.276, or 0 W.

For MoS_2_‐TFT and OLED integration, the parameters for calculating the transistor‐OLED integration are defined according to the reference:^[^ ^38^ ^]^ *W* = 300 µm, *L* = 4 µm, *d* = 0.1 µm, *S* = 0.09 × 0.09 = 0.0081 cm^2^, *C*_i_ = 1.6  ×  10^–7^ F cm^‐2^ (50 nm Al_2_O_3_ with *ε*_ox_ = 9*ε*_0_), *µ*_1_ = 18 cm^2^ V^‐1^ s^‐1^ (MoS_2_)*, V*_th_ = 4 V, *µ*_2_ = 0.0005 cm^2^ V^‐1^ s^‐1^ (OLED), and *V*_0_ = 3 V. Then, the *I–V* characteristics are calculated by Equations (5 and 7) with the factors as *α* = 2 and *Q*_0_= 3  ×  10^–13^ C cm^‐3^ (ideal SCLC and *ε*_SC_ = 3*ε*_0_ for organic semiconductors).

For Si‐MOSFET and ZnO‐emitter integration, the field emission current of the ZnO nanowire in the integrated device was measured while they were being controlled by the Si‐MOSFET. The integrated device was placed in a high‐vacuum chamber (5 × 10^−5^ Pa). The anode current was probed by a monitored with a stainless‐steel probe (1 mm diameter) and biased by a power supply picoammeter (Keithley 6487).

### Device Calculation

A calculator for different devices is compiled in an open‐source, HTML file with a user interface for demonstration and testing in the Supporting Information. Readers may open the “index” file to use it and read the “Manual” file for assistance. The calculator was tested by comparing some results with those obtained from TCAD simulations with varied device or material parameters. The calculations and fittings in this manuscript could be obtained by setting the corresponding parameters in this calculator. The calculator could be used to fit the experimental data or predict performance of some transistors. When calculating the split‐gate transistors, the device has the dimension of *L*_1_ = 8 µm, *d* = 2 µm, and *L*_2_ = 8 µm, with other parameters as the same as those in the drain‐offset transistors.

## Conflict of Interest

The authors declare no conflict of interest.

## Supporting information

Supporting Information2

Supporting Information2

## Data Availability

The data that support the findings of this study are available from the corresponding author upon reasonable request.
