# Supplementary material for: How Materials and Device Factors Determine the Performance: A Unified Solution for Transistors with Nontrivial Gates and Transistor–Diode Hybrid Integration
Source: Adv Sci (Weinh). 2021 Dec 16;9(5):2104896. doi: 10.1002/advs.202104896 (PMC8844558; doi:10.1002/advs.202104896)
Supplement: Supplementary file 1 — Supporting Information2 [file ADVS-9-2104896-s002.pdf]

ADVANCED SCIENCE
Open Access

Supporting Information

for *Adv. Sci.*, DOI: 10.1002/advs.202104896

How Materials and Device Factors Determine the Performance: A Unified Solution for Transistors with Nontrivial Gates and Transistor-Diode Hybrid Integration

Chuan Liu, Xiaojie Li, Yiyang Luo, Ya Wang, Sujuan Hu, Chenning Liu, Xiaoci Liang, Hang Zhou, Jun Chen, Juncong She, Shaozhi Deng*

Supporting Information

Figure S1. Parameters of α and Q_0 of the devices with varied donor-like or acceptor-like states.
Figure S2. The extracted $V_{D,SAT}$ (dots) are plotted against $(V_G - V_{th})$, $1/t_{ox}{}^{0.5}$, and $d^{1.5}/L^{0.5}$, respectively.
Figure S3. Electric potential near the semiconductor-dielectric interface for devices with varied t_{SC} or t_{ox}.
Figure S4. The user interface of the index in the HTML file for calculating transistors.
Figure S5. The user interface of the HTML file for calculating drain-offset transistors.
Table S1. The TCAD simulation parameters of top-contact, bottom-gate transistors.
Table S2. The parameters for *I-V* curves with donors or acceptors.
Table S3. The set parameters (N_D and w_A) and fitting parameters (α, V_0, Q_0) for devices with donor-like states or acceptor-like states.
Supporting Note 1. The *space-charge-to-equilibrium-carrier ratio* η.
Supporting Note 2. Equation derivation of general trap-limited SCLC.
Supporting Note 3. F-N tunneling or other conduction mechanisms.
Supporting Note 4. Solving drain-offset transistors.
Supporting Note 5. Depletion region width.
Supporting Note 6. Solving split-gate transistors.
Supporting Note 7. Solving mid-gate transistors and static induction transistors.

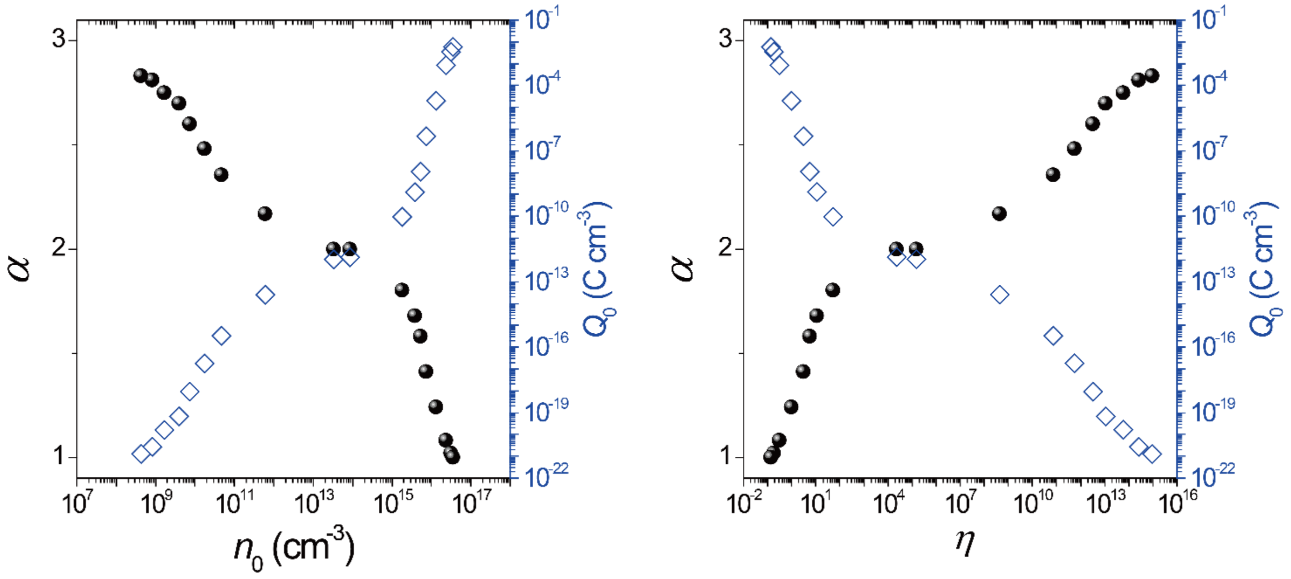

Figure S1. Parameters of α and Q_0 of the simulated devices with varied donor-like or acceptor-like states, as a function of (a) n_0 and (b) calculated η (V_G- V_{th}= 3 V, C_i = 6.9×10^{-8} F/cm^2, t_{SC} = 20 nm, V_1 = 1.5 V). The values of α and Q_0 are obtained by using Eq. (5) to the *I-V* data of simulated devices with varied density of donor-like or acceptor-like states, i.e., varying N_D from 10^{16} to 10^{18} cm^{-3} with fixed at 0.4 eV, or varying N_A from 5×10^{16} to 5×10^{18} cm^{-3} at fixed w_A fixed at 0.1 eV. In particular, when N_D = 10^{18} cm^{-3}, n_0 is 3.6×10^{16} cm^{-3}, qn_0 is 5.7×10^{-3} C/cm^3, and Q_0 is 5.9×10^{-3} C/cm^3, consistent with the predicted relation $Q_0{\sim}qn_0$ in Ohmic conduction. When N_D = 0 cm^{-3}, n_0 is 8.5×10^{13} cm^{-3}, $9\varepsilon_{sc}/8$ is 1.0×10^{-12}, and Q_0 is 1.3×10^{-12} C/cm^3, consistent with the predicted relation $Q_0{\sim}9\varepsilon_{sc}/8$ with SCLC in the non-gated channel. The general trend in (b) is presented in Figure 3a in the main context.

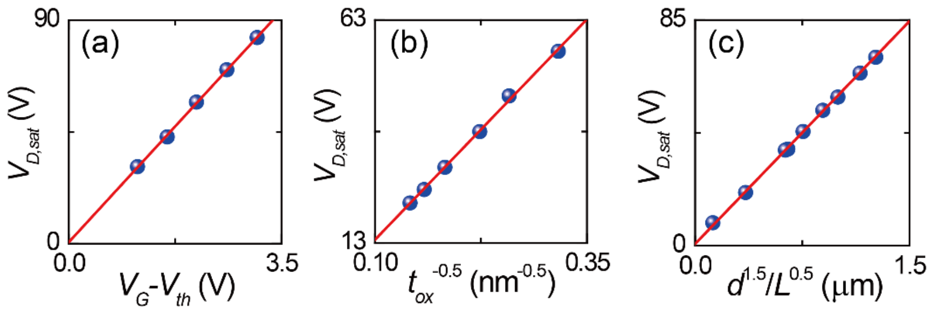

Figure S2. The extracted $V_{D,SAT}$ from device simulation (dots) are plotted against $(V_G - V_{th})$, $1/t_{ox}{}^{0.5}$, and $d^{1.5}/L^{0.5}$, respectively. The saturation voltages corresponding to the ideal SCLC (Figure 5a-c), the parameters for the TCAD simulation are the same as list in the main context and the corresponding parameters are varied, including V_G, L, d, and t_{ox}. The extracted $V_{D,SAT}$ of devices with varied parameters (V_D, t_{ox}, d, and L) are found to increase linearly with (V_G-V_{th}), $1/t_{ox}{}^{0.5}$, and $d^{1.5}/L^{0.5}$, corresponding to Eq. (9) with SCLC. The same data are plotted together in Figure 5d in the main context.

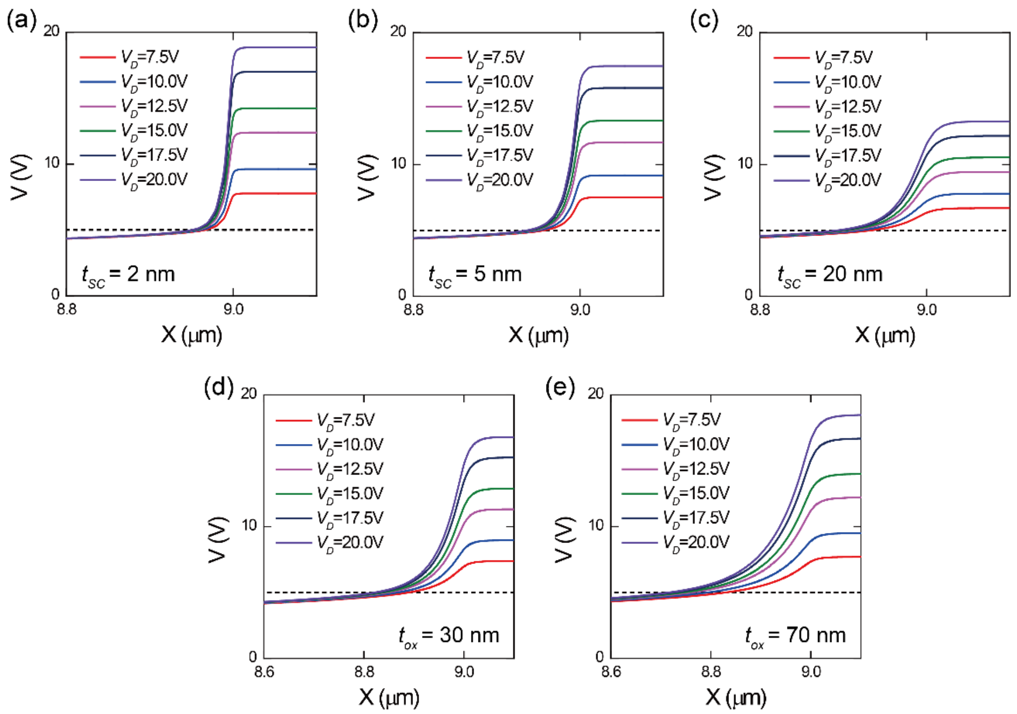

Figure S3. Electric potential near the semiconductor-dielectric interface for devices with varied t_{SC} or t_{ox} by device simulation. In (a-c), t_{SC} is varied from 2, 5, to 20 nm with t_{ox} fixed at 10 nm. In (c-e), t_{ox} is varied from 10, 30, to 20 nm with t_{SC} fixed at 20 nm. The gate is from X = 1 to 9 μm and the drain is from X = 9 to 10 μm. ΔL is the distance from the position where the interfacial potential reaches V_G - V_{th} (5 V, dashed line) to the drain. The extracted values of ΔL are plotted in Figure 5e in the main context.

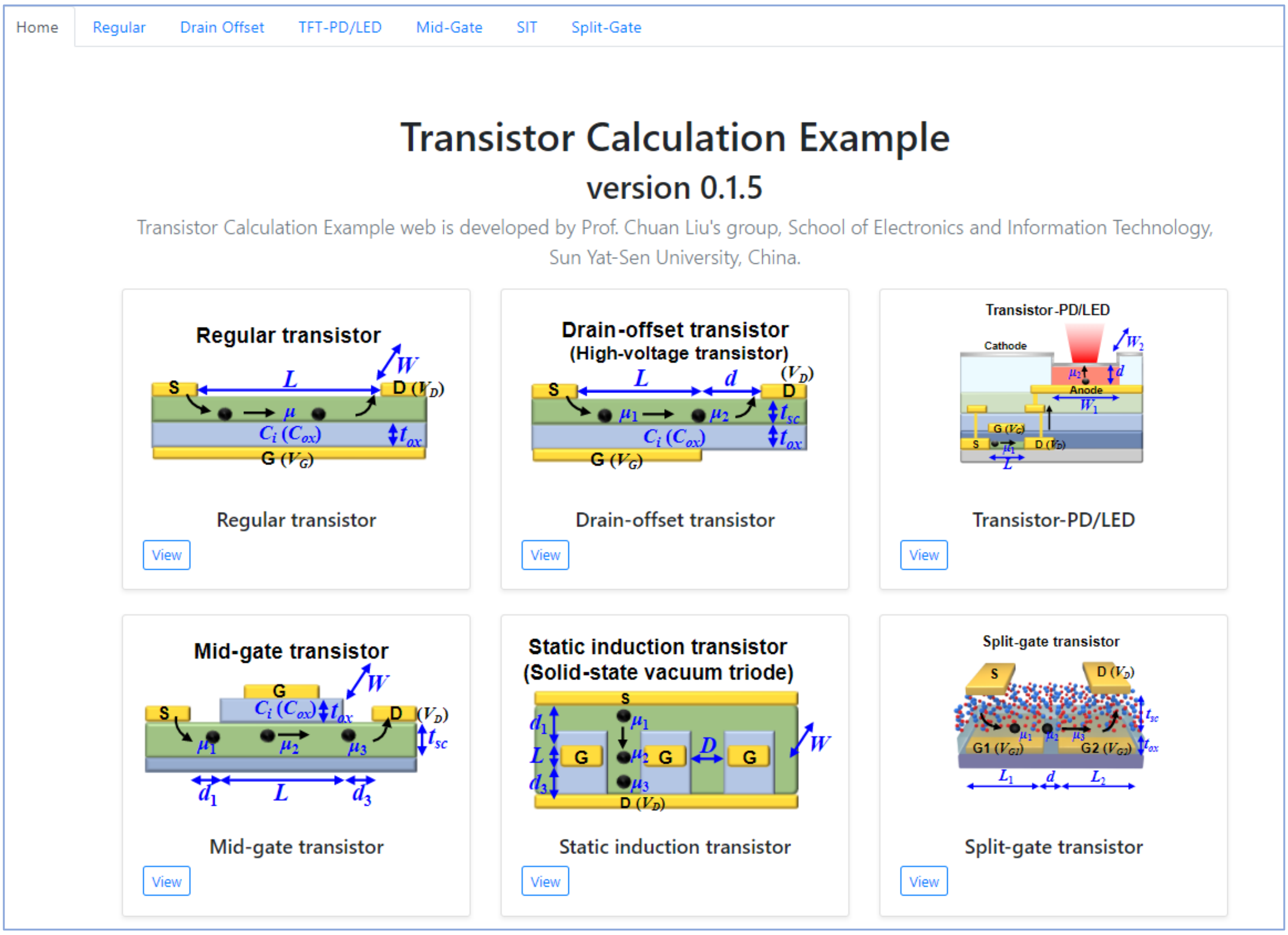

Figure S4. The user interface of the index in the HTML file for calculating transistors. It is contained in the supplementary information.

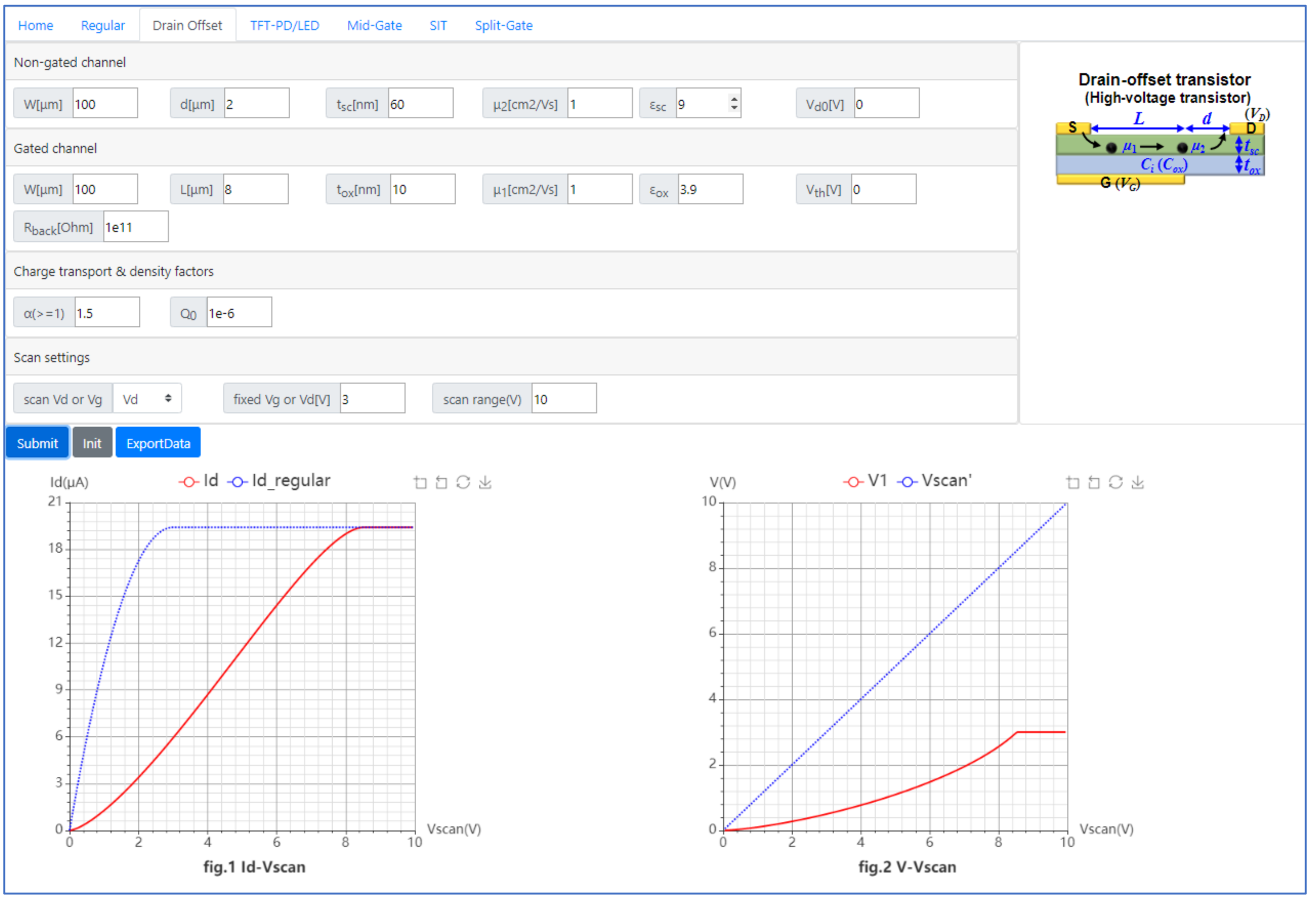

Figure S5. The user interface of the HTML file for calculating drain-offset transistors. The user interfaces for calculating other transistors are similar.

Table S1. The TCAD simulations of top-contact, bottom-gate transistors were performed by the SILVACO platform and the main parameters for material definition is as follows. Electric breakdown is not considered for simplicity. The basic device parameters are W = 1000 μm, L = 8 μm, d = 2 μm, t_{sc} = 20 nm, $\mu_1 = \mu_2$ = 1 cm^2/Vs, and C_i = 11 nF cm^{-2}.

**Category**	**Parameters**	**Values or materials**
Semiconductor	Electron affinity / Ionization potential (eV)	4.3 / 3.3
	Mobility (cm^2/Vs)	1 (*n*-type)
	Relative permittivity ε	9
	Thickness of semiconductor (nm)	20 (or 2, 5)
	Channel region (μm) (when L = 8 and d = 2)	X=1 to 9 (or other values)<br>Y = 0 to 0.02 (or other values)
Electrodes	Work-function of source/drain (eV)	4.3
	Work-function of gate (eV)	4.3
	Source and drain region (μm) (when L = 8 and d = 2)	X=0-1 and X=11-12 (or others)<br>Y = 0

	Gate region (μm) (when L = 8 and d = 2)	X=0-9
	Thickness of source/drain/gate (nm)	0
Dielectric	Relative permittivity ε_{ox} and bandgap (eV)	3.9 and 9.0
	Thickness t_{ox} (nm)	10 (or 5, 20, 30, 40, 50, 70)
	Dielectric region (μm)	X=0 to 10; Y = 0.02 to 0.03 (or other values)
Geometry	Width W (μm)	1000
	Gated channel length L (μm)	8 (or 4, 10, 12)
	Non-gated channel length d (μm)	2 (or 1, 3, 4)

Table S2. For *I-V* curves with donors or acceptors (Figure 3 in the main context), the parameters for devices with donor- or acceptor-levels.

Gaussian distributed, donor-like states		Exponentially distributed, acceptor-like states	
Peak number N_D (10^{17} cm^{-3}eV^{-1})	0, 1, 2, or 5	Peak number N_A (10^{18} cm^{-3}eV^{-1})	1
Energy level E_D (eV) (referencing to the electron affinity)	0.4	Width of the tail states w_A (eV)	0, 0.08, 0.10, or 0.15
Width of Gaussian distribution w_D (eV)	0.1	-	-

Table S3. The set parameters (N_D and w_A) and fitting parameters (α, V_0, Q_0) for devices with donor-like states or acceptor-like states for Figure 3 in the main context. The curve fittings were performed by first fitting the value of α (and V_0 if necessary) and then estimating the values of Q_0 as: $Q_0 \approx I_D d^\beta/[S\mu_2(V_D - V_{D0})^\alpha]$ with a certain V_D value (but is generally applicable for all the V_D values). The values of γ are calculated as $\gamma = (LSQ_0\mu_2)/(Wd^\beta C_i\mu_1)$ if needed. Here, the value of β is set as 2α-1.

N_D (10^{17} cm^{-3}eV^{-1})	α	V_0 (V)	Q_0 (C/cm^3)	w_A (eV)	α	V_0 (V)	Q_0 (C/cm^3)
0	2.00	0.0	1.3×10^{-12}	0	2.00	0.0	1.3×10^{-12}
1	1.41	0.0	4.6×10^{-7}	0.08	2.17	0.0	2.6×10^{-14}
2	1.24	0.0	2.0×10^{-5}	0.10	2.36	0.5	3.4×10^{-16}
5	1.08	0.0	8.2×10^{-4}	0.15	2.70	9.0	6.8×10^{-20}

Supporting Note 1

The *space-charge-to-equilibrium-carrier ratio* η. The n_{Sp} responsible for forming space charge regions will increase when increasing the *dielectric relaxation time* τ_R within which carriers are relaxed toward a uniform distribution, decreasing the *carrier transit time* τ_T within which carriers are swept out, or increasing the injected carrier density n_{inj}. We have $\eta = n_{Sp}/n_0 = n_{inj}n_0/\tau_R\tau_T$, where $\tau_T \approx d^2/(\mu\Delta V)$ with the voltage drop $\Delta V = V_2 - V_1$ and $\tau_R = \varepsilon_{sc}/(n_0 q\mu)$.

For n_{inj} denoting the density of carriers forming space charge regions, we could consider that: the carriers injected from the gated semiconductor will reach the end of the non-gated semiconductor within the transit time τ_T, therefore giving the total charges within the non-gated semiconductor as $I_D\tau_T$. Then, the average density of the injected carriers distributed within the volume of the non-gated semiconductor are

characterized by $I_D\tau_T/(qSd)$, where S is the current area and d is the transit length. Therefore, the average value of η can be characterized as:

$$\eta = \frac{n_{Sp}}{n_0} = \frac{n_{inj}}{n_0}\cdot\frac{\tau_R}{\tau_T} \approx \frac{I_D\tau_T}{qn_0Sd}\cdot\frac{\tau_R}{\tau_T} = \frac{W\mu_1C_i(V_G-V_{th}-V_1/2)V_1}{Lqn_0Sd}\cdot\frac{\varepsilon_{sc}}{n_0q\mu_2} \approx \frac{WC_i(V_G-V_{th})V_1\varepsilon_{sc}}{LSd(qn_0)^2}\frac{\mu_1}{\mu_2}. \quad \text{(s1)}$$

Here, ε_{sc} is the permittivity of the non-gated semiconductor, and μ_1 and μ_2 are the carrier mobility in the gated and non-gated semiconductors. V_1 is the potential at the end of the gated-channel (with the upper limit V_G-V_{th} or V_D). In a drain-offset transistor, $S = Wt_{sc}$ with t_{sc} as the thickness of the non-gated semiconductor. The value of η increases if V_1 increases. In the main context, $V_G - V_{th} - V_1/2$ is approximated as $V_G - V_{th}$ for simplicity. In practice, when estimating the value of η, $V_1 = (V_G - V_{th})/2$ could be used.

Supporting Note 2

Equation derivation of trap-limited SCLC. The current density is assumed to be:

$$J = n(n/n_{t0})^{\delta-1}q\mu_0\epsilon(\epsilon/\epsilon_{t0})^{\kappa-1} = An^{\delta}q\mu_0\epsilon^{\kappa} \quad \text{(s1)}$$

Here, n is the carrier density, q is the elementary charge, μ_0 is characteristic carrier mobility, ϵ is the drift electric field, n_{t0} and ϵ_{t0} are characteristic carrier density and drift electric field mainly related to bulk traps of interfacial states, δ and κ are power factors, and $A = (1/n_c)^{\delta-1}(1/\epsilon_c)^{\kappa-1}$. Rewrite Eq. (s1) into $n = \sqrt[\delta]{J/(Aq\mu_0\epsilon^{\kappa})}$, substitute it into Poisson equation $d\epsilon/dx = nq/\varepsilon$, integrate the field $d\epsilon$ and distance dx, and solve the electric field:

$$|\epsilon| = \left[\frac{\delta+\kappa}{\delta}\frac{q}{\varepsilon}\left(\frac{J}{Aq\mu_0}\right)^{\frac{1}{\delta}}x\right]^{\frac{\delta}{\delta+\kappa}} \quad \text{(s2)}$$

Integrate through distances d and the potential drop is:

$$V = \int_0^X|\epsilon|dx = \left[\frac{\delta+\kappa}{\delta}\frac{q}{\varepsilon}\left(\frac{J}{Aq\mu_0}\right)^{\frac{1}{\delta}}\right]^{\frac{\delta}{\delta+\kappa}}\frac{\delta+\kappa}{2\delta+\kappa}d^{\frac{2\delta+\kappa}{\delta+\kappa}} \quad \text{(s3)}$$

Rearrange Eq. (s3) and the current-voltage relation is:

$$J = A\left(\frac{\delta}{\kappa+\delta}\right)^{\delta}\left(\frac{\kappa+2\delta}{\kappa+\delta}\right)^{\kappa+\delta}\frac{\varepsilon^{\delta}}{q^{\delta-1}}\mu_0\frac{V^{\kappa+\delta}}{d^{\kappa+2\delta}} = Q_0\mu_0\frac{V^{\alpha}}{d^{\beta}} \quad \text{(s4)}$$

Here, $\alpha = \kappa + \delta$ and $\beta = \kappa + 2\delta$.

Supporting Note 3

F-N tunneling or other conduction mechanisms. In F-N tunneling, the current density is simply expressed as $J{\sim}V^2\exp(-V_a/V)$, where V_a is a constant. When V is large, we could use the 2nd order Taylor expansion for approximation: $J{\sim}V^2\exp(-V_a/V) \cong V^2(1 - V_a/V + {V_a}^2/2V^2) \propto (V - V_a)^{\alpha}$, where $\alpha \approx 2$. Therefore, for F-N tunneling, we could use Eq. (4) with $\alpha \approx 2$. Similarly, for other conduction mechanisms, e.g., thermionic emission, hopping conduction, P-F emissions, and etc., if the power law dependence of J-V is applicable, Eq. (4) could be used as an approximation for an on-state device. In general, if the current density could be described as $J{\sim}V^a\exp(KV^b)$ ($b > 0$) it could be approximated as: $J{\sim}V^a\exp(KV^b) \approx V^a(1 + KV^b + K^2V^{2b}/2) = V^a[(KV^b + 1)^2 + 1]/2 \propto K^2V^{a+2b}/2$. In P-F emission, $a = 1$ and $b = 1/2$. Hence, the power law dependence of J-V could be a good approximation within a certain range of operational window.

Supporting Note 4

Drain-offset transistors. In Eq. (6) in the main context, the 2nd order Taylor series is applied to the right as $V_1 < V_D$. Then we have $\gamma V_D^{\alpha}(1-V_1/V_D)^{\alpha} = \gamma V_D^{\alpha}[1-\alpha V_1/V_D+\alpha(\alpha-1)(V_1/V_D)^2/2] = \gamma V_D^{\alpha}-\alpha\gamma V_1 V_D^{\alpha-1}+\gamma\alpha(\alpha-1)V_1^2 V_D^{\alpha-2}/2$. Rearrange Eq. (7) and we have $aV_1^2+bV_1+c=0$, where $a=1+\gamma\alpha(\alpha-1)V_D^{\alpha-2}$, $b=-2(V_G-V_{th}+\alpha\gamma V_D^{\alpha-1})$, and $c=2\gamma V_D^{\alpha}$. The accurate solution is: $V_1=\left(-b-\sqrt{b^2-4ac}\right)/(2a)$. In particular, when γ is small, $V_1 \ll V_D$ and the right of Eq. (7) becomes γV_D^{α}. Therefore, the approximate solution is: $V_1 \approx (V_G-V_{th})-\sqrt{(V_G-V_{th})^2-2\gamma V_D^{\alpha}}$. In calculation, the upper limit of $|V_1|$ is the minimum of $|V_G-V_{th}|$ and $|V_D|$.

The maximum electric field in the non-gated channel E_{MAX} is obtained when $x = L + d$: $|E_{MAX}| = |E_X(x=L+d)| = \beta|\Delta V|/(\alpha d)$. For a semiconductor with the breakdown field E_B, we have $|E_{MAX}| \leq E_B$ and so the maximum voltage $|\Delta V|_{max}$ across the non-gated semiconductor is $|\Delta V|_{max}=\alpha d E_B/\beta$. The maximum current is:

$$I_{D,max}=Q_0 S\mu_2\frac{(\Delta V_{max})^{\alpha}}{d^{\beta}}=Q_0 S\mu_2\frac{(\alpha E_B)^{\alpha}}{\beta^{\alpha}d^{\beta-\alpha}} \quad \text{(s5)}$$

The maximum virtual power is:

$$P_{max}=V_{D,max}I_{D,max}=Q_0 S\mu_2\frac{(\alpha E_B)^{\alpha+1}}{\beta^{\alpha+1}d^{\beta-\alpha-1}}\propto {E_B}^{\alpha+1} \quad \text{(s6)}$$

The local power density for Joule heating in the non-gated channel is calculated by using Eq. (8):

$$p(x)=J|E_X(x)|=\frac{I_{D,SAT}}{S}\frac{\beta(V_D-V_G+V_{th})}{\alpha d^{\beta/\alpha}}(x-L)^{\beta/\alpha-1} \quad \text{(s7)}$$

The maximum value is obtained at $x = L$. Here, $I_{D,SAT}$ is the saturated current: $I_{D,SAT}=W\mu_1 C_i(V_G-V_{th})^2/(2L)$.

Supporting Note 5

Depletion region width. For Figure 5e in the main context, the device parameters are W = 1000 μm, L = 8 μm, d = 2 μm, t_{sc} = 20 nm, $\mu_1=\mu_2$ = 1 cm^2/Vs, and C_i = 11 nF cm^{-2}. The bias condition is V_G = 5 V and V_D is from 7.5 V to 20 V (step 2.5 V). For the first three series of dots in Figure 5e, t_{SC} is 2 nm, 5 nm, or 20 nm (with t_{ox} fixed at 10 nm). For the second three series of dots in Figure 5e, t_{ox} is 10 nm, 30 nm, or 70 nm (with t_{SC} fixed at 20 nm). ΔL is the distance from the position where the interfacial potential reaches V_G - V_{th} (5 V) to the drain. The channel potential of simulated devices is shown in Supporting Fig. 3.

Also, the TCAD simulation data show that Eq. (10) in the main context could be used to estimate ΔL by using α = 1.9 when $V_D/(V_G-V_{th}) < 3$. For example, when t_{SC} = 20 nm and t_{ox} = 10 nm, the estimated ΔL by Eq. (10) is 88 nm and the TCAD simulated ΔL is 78 nm for V_D = 10 V. According to Eq. (10), the channel length modulation effect is characterized by:

$$\frac{L}{L-\Delta L}=\frac{L}{L-\sqrt[3]{fL\lambda^2}}=\frac{1}{1-\sqrt[3]{f(\lambda/L)^2}}. \quad \text{(s8)}$$

Here, f is a function of $V_D/(V_G-V_{th})$. Hence, $L/(L-\Delta L)$ will increase if L increases, but will decrease if λ decreases.

We could use Eq. (s7) for regular TFTs by replacing d by ΔL:

$$p_{max}=p(x=L)=\frac{\beta}{\alpha S\Delta L}\left|(V_D-V_G+V_{th})I_{D,SAT}\right|\approx\frac{3}{2S\Delta L}\left|(V_D-V_G+V_{th})I_{D,SAT}\right|, \quad (11)$$

It indicates that Joule heating in a saturated transistor is more severe in short-L devices even with the same J.

Supporting Note 6

Split-gate transistors. A split-gate transistor could be regarded as a *Gated/Non-gated/Gated* structure. Denote $V_{Gt1} = V_{G1} - V_{th1}$ (near the source) and $V_{Gt2} = V_{G2} - V_{th2}$ (near the drain) and, for generality, the current is:

$$I_D = \frac{W_1}{L_1}\mu_1 C_i\left(V_{Gt1} - \frac{V_1+V_S}{2}\right)(V_1 - V_S) = SQ_0\mu_2\frac{(V_2-V_1)^\alpha}{d^\beta} = \frac{W_3}{L_3}\mu_3 C_i\left(V_{Gt2} - \frac{V_D+V_2}{2}\right)(V_D - V_2) \quad \text{(s11)}$$

Usually, $W_1 = W_3$ and $\mu_1 = \mu_3$. Denote $\gamma_{12} = (S\mu_2 Q_0 L_1)/(W_1\mu_1 C_i d^\beta)$, $\gamma_{32} = (S\mu_2 Q_0 L_3)/(W_3\mu_3 C_\mathrm{i} d^\beta)$, $\gamma_{31} = (W_1\mu_1 L_3)/(W_3\mu_3 L_1)$, and $\Delta V = V_2 - V_1$. Assume $V_S \approx 0$ (Ohmic contact) so that Eq. (s11) is simplified as:

$$\frac{1}{\gamma_{12}}\left(V_{Gt1} - \frac{V_1}{2}\right)V_1 = \Delta V^\alpha = \frac{1}{\gamma_{32}}\left(V_{Gt2} - \frac{V_D+V_2}{2}\right)(V_D - V_2) \quad \text{(s12)}$$

We can simplify the problem when scanning V_{G1}: (1) Treat the 2nd (non-gated) and 3rd (gated) channels as a whole and calculate V_1 by the same method in the main texts with a V_{G2}-dependent Q_0 (i.e., $Q_{00}V_{Gt2}^r$, r is the power of Q_0); (2) Use V_1 to calculate the corresponding V_2 by equaling the 1st and 3rd term in Eq. (s12); (3) As the 3rd (gated) channel with a fixed V_{G2} limits the current, the maximum $V_{1,max}$ can be calculated by equaling V_1 and V_2 in Eq. (s12). Notice that the upper limit of V_1 is the minimum of $V_{1,max}$, V_{Gt1}, V_{Gt2}, and V_D, whereas the upper limit of V_2 is V_{Gt2} and V_D. Such asymmetry is caused by the asymmetry of source and drain voltages. The calculated results are consistent with those from TCAD, indicating the simplification reveals the key physics. Readers could use the attached HTML file for practical calculations or fittings, including transfer or output characteristics.

When calculating the split-gate transistors (Fig. 7 in the main context), the device has the dimension of L_1 = 8 μm, d = 2 μm, and L_2 = 8 μm, with other parameters as the same as those in the drain-offset transistors.

Supporting Note 7

Mid-gate transistors and static induction transistors. Generally, the *Non-gated/Gated/Non-gated* structure could be described by modifying Eq. (5) as:

$$I_D = Q_{01}S_1\mu_1\frac{(V_1-V_S)^\alpha}{d_1^\beta} = \frac{W}{L}\mu_2 C_i\left(V_G - V_{th} - \frac{V_2+V_1}{2}\right)(V_2 - V_1) = Q_{03}S_3\mu_3\frac{(V_D-V_2)^\alpha}{d_3^\beta}. \quad \text{(s9)}$$

Here, V_1 and V_2 are the potential at the beginning and end of the gated channel, and S_n, d_n, μ_n and Q_{0n} are the area, length, carrier mobility, and charge density factors for the nth channel, respectively. Denote $\gamma_{12} = (S_1\mu_1 Q_{01} L)/(W\mu_2 C_\mathrm{i} d_1^\beta)$, $\gamma_{23} = (S_3\mu_3 Q_{03} L)/(W\mu_2 C_\mathrm{i} d_3^\beta)$, $\gamma_{31} = (Q_{03}S_3\mu_3 L_1^\beta)/(Q_{01}S_1\mu_1 L_3^\beta)$, and $V_{Gt} = V_G - V_{th}$. Eq. (s8) could be used to solve mid-gate transistors and static induction transistors.

As planar, mid-gate transistors are usually made of symmetric non-gated channels, we could use $V_S \approx 0$, $\mu_1 = \mu_2 = \mu_3$, $S_1 = S_3$, $d_1 = d_3$, $\gamma_{12} = \gamma_{23} = \gamma = (S_3 Q_{03} L)/(W C_\mathrm{i} d_3^\beta)$ and $\gamma_{31} = 1$. Then, in the *unsaturated* regime, Eq. (s9) turns into:

$$\gamma V_1^\alpha = \left(V_{Gt} - \frac{V_2+V_1}{2}\right)(V_2 - V_1) = \gamma(V_D - V_2)^\alpha. \quad \text{(s10)}$$

Use the 2nd order Taylor expansion on the last term and we have $a_2V_2^2 + b_2V_2 + c_2 = 0$, where $a_2 = \gamma\alpha(\alpha-1)V_D^{\alpha-2}/2$, $b_2 = -(\gamma\alpha V_D^{\alpha-1} + 2V_{Gt} - V_D)$, and $c_2 = \gamma V_D^\alpha + (V_{Gt} - V_D/2)V_D$. The potential V_2 with the upper limit V_{Gt} and $V_1 = V_D - V_2$ could be solved. The *saturated* regime could be solved similarly. With the values of V_1 and V_2, the *I*-*V* relations could be obtained from Eq. (s9). Readers could use the attached HTML file for calculations or fittings, including transfer or output characteristics. For mid-gate transistors used for high power or logic applications, the semiconductors are usually conductive to make Ohmic contacts and the value

of α should be close to 1.

For static induction transistors, solid-state vertical triodes, or vertical transistors with permeable gate electrodes, we usually have $\gamma_{12} \neq \gamma_{23}$ and Eq. (s9) is rewritten to be $a_3 V_2^2 + b_3 V_2 + c_3 = 0$, where $a_3 = \gamma_{32}\alpha(\alpha-1)V_D^{\alpha-2}/2 - (\sigma^2 - 1)/2$, $b_3 = -\gamma_{32}\alpha V_D^{\alpha-1} - (V_{Gt} - \sigma V_D/2)(\sigma+1) + (\sigma-1)\sigma V_D/2$, $c_3 = \gamma_{32}V_D^{\alpha} + (V_{Gt} - \sigma V_D/2)\sigma V_D$, and $\sigma = \sqrt[\alpha]{\gamma_{31}}$. The potential V_2 and V_1 could be solved as the same above. Also, another current path directly from the top source to the bottom drain should be taken into account, where SCLC is assumed by calculating its resistance as $R_{MID} = \rho_{MID}(d_1 + L + d_2)/D$. Here, ρ_{MID} is the resistivity for the SCLC [$\rho_{MID} = (1/V_D)\rho_{MID0}$] and D is the diameter of the hole, as shown in the calculator file. Readers could use the attached HTML file for calculations. Since the semiconductors are usually intrinsic to give a high on-off ratio, the value of α is recommended to be above 1.
